# Another Step in Higher Medical Education

**Published:** 1917-01

**Authors:** 


### ANOTHER STEP IN HIGHER MEDICAL EDUCATION.

Among the remarkable developments in medicine anywhere, at any time, are the changes that have occurred in medical education in the United States during the last ten or fifteen years. Once regarded as a disgrace, the standard of medical education in this country recently has advanced so rapidly that today it is equal to that of any other nation so far, at least, as the majority of medical teaching institutions are concerned. This change has occurred partly through the generosity of wealthy men and women who have contributed millions to medical schools and to medical research; partly because the medical profession itself became aroused to the wretched conditions in medical education, and undertook to rid itself of the incubus of the purely commercial medical school. Both were necessary to bring about the rapid advances that have placed American medicine in the high position it now occupies.

The announcement just made of the establishment, as a department of the University of Chicago, of a new medical school, complete with post-graduate departments, extensive hospital facilities, numerous research branches, with a standard as high as that of any medical school here or abroad, and with an endowment sufficient to meet the expenses connected with full-time, paid instructors in all departments, is one of the most important events connected with the rapid development of scientific medicine and medical teaching in this country. It means much not only to medical education, but, more important, to public health, for it will be not only a teaching institution—an institution that will make for better and more broadly and practically educated medical practitioners—but also an institution for the development of preventive medicine. It means much to the city of Chicago—once the home of more quack medical colleges and diploma mills than any other city in the world. It will aid the city, which was the plague spot of medical education, to rid itself wholly of the commercial school and to develop medical institutions second to none. But the influence of this new institution will be broader than the city or the state in which it is located. It will be national, and will reach out and stimulate good work in every part of the country.

To President Harry Pratt Judson, of the University of Chicago, and to Dr. Frank Billings, Dean of Rush Medical College, is due, to a large extent, the credit for this achievement. It means the successful culmination of President Judson's ambition to carry out not simply the original plan of his predecessor—once looked on as a dream—but a greater and broader one than even President William Rainey Harper conceived.—*Journal A. M. A.*

